# Learning from Gobind Khorana: “The difficult we do today, the impossible tomorrow”

**DOI:** 10.1007/s12551-022-01019-4

**Published:** 2022-11-30

**Authors:** Sriram Subramaniam

**Affiliations:** Department of Biochemistry and Molecular Biology, University of British Columbia, Vancouver, BC Canada

**Keywords:** Bacteriorhodopsin, Gene synthesis, Genetic code, Rhodopsin, Chemical biology

## Abstract

Prof. Har Gobind Khorana was one of the greatest scientists of the twentieth century. Drawing on his strong roots in organic chemistry, he had a remarkable ability to select and focus his intellect on successfully addressing some of the most important challenges in modern biology in a career spanning nearly 6 decades. His pioneering contributions in gene synthesis and protein structure–function studies, and more broadly in what he termed “chemical biology,” continue to have a major impact on modern biomedical science.

The print on the note pasted on the refrigerator door was beginning to fade and the Scotch tape was loose at the edges, but the contents are etched forever in my memory. It had just these eight words: “The difficult we do today, the impossible tomorrow.”

I arrived at MIT in the first week of April 1987 to begin my postdoctoral fellowship in Gobind’s lab. My 5-ft-long bench portion was largely empty, save for some buffers and pipette boxes that had been left behind by the previous occupant. There was a shared refrigerator nearby with shelves and column purification equipment. The first thing that caught my eye as I was looking around was the pasted piece of paper with those eight words. I did not know the origin of the quote, which was not attributed to anyone, nor do I recall that Gobind actually said these words to me during my time in the lab, but I cannot think of a better summary of his scientific approach and philosophy. The absolute standards and rigor that were at the core of Gobind’s science left an indelible impact on all of us who had the privilege to work with him directly.

Gobind was recruited to the University of British Columbia by Gordon Shrum in the 1950s to help build up organic chemistry research as it related to interests at the University at the time. His lab was in a small space at one end of the campus, where he initiated his studies on biologically important organo-phosphates. Legend has it that to characterize the purified nucleotide fractions emerging from the column, Gobind would regularly race back and forth with test tube in hand to a building some distance away that had a spectrophotometer to measure the optical density. Within a couple of years, Arthur Kornberg and Paul Berg made trips to Vancouver from Stanford to learn and implement the methods Gobind was developing in his modest laboratory. The extraordinary scientific revolution that Gobind and his student Michael Smith led at the interface between chemistry and biology laid the early foundations both for Gobind’s 1968 Nobel prize and Michael Smith’s 1993 Nobel prize. Gordon Shrum’s decision to recruit Gobind changed the world of molecular biology forever.

Over the years, Gobind trained over hundreds of scientists, young and old, over the course of his career at the University of British Columbia, the University of Wisconsin at Madison, and MIT. His style of teaching was by example. He never preached, but his scientific discipline was a powerful beacon that guided everyone in his orbit. The decades-long partnership that Gobind had with Prof. Tom Rajbhandary was a critical element of the scientific atmosphere for members of both laboratories. Tom complemented Gobind in many respects and shared the great love of rigorous science that was the hallmark of the training they imparted. Regular tri-weekly lab meetings were jointly held with the students and postdocs in both laboratories, and this led to much greater intellectual breadth for all of us who benefited from this arrangement. The atmosphere was a magnet for attracting trainees who came from all over the world: I was especially fortunate to be in the company of wonderful individuals such as Sadu Karnik, Dan Oprian, Tom Sakmar, Roland Franke, Larry Stern, Tomoko Doi, and Duncan Greenhalgh and so many others who have all gone on to illustrious careers after leaving MIT.

Most of my training with Gobind was in the context of his work on bacteriorhodopsin, which, for me and others in the field, was a master class in how great scientists define and tame grand challenges in biology. Gobind was a legend already in the nucleotide field and had carried out ground-breaking work on total gene synthesis. But his quest to dig deeper into biology at the molecular level led him to the world of proteins where he himself did not have much direct experience, nor were there clear paths that had been laid by others for the systematic dissection of chemical mechanisms underlying protein function.

By the early 1980s, bacteriorhodopsin was one of the very few membrane proteins that had been studied in depth by biophysicists, in large part because it was a protein with a chromophore. The presence of the chromophore retinal, which was covalently attached to the protein, made it tractable to analysis using a host of spectroscopic methods especially including optical and vibrational spectroscopy. The protein itself was originally discovered by Walther Stoeckenius and co-workers in the late 1960s in the salt-loving organism *Halobacterium Halobium*, and spawned the careers of many scientists who saw the protein as a tool to better understand the mammalian visual pigment rhodopsin, which also has the same chromophore retinal bound covalently to the protein. For many scientists, another major impetus to study bacteriorhodopsin was that Nigel Unwin and Richard Henderson had reported in 1975 that electron beams could be used to study the three-dimensional structure of bacteriorhodopsin at a resolution of ~ 7 Å, a stunning advance in structural biology at the time.

Gobind’s interest was not centered in the structure or the spectroscopy of bacteriorhodopsin, but rather in the chemistry and mechanism by which the protein worked. Bacteriorhodopsin is a light-driven proton pump and was of considerable interest in the general context of energy metabolism, especially because of the Peter Mitchell’s chemiosmotic hypothesis connecting proton gradients and ATP synthesis. Gobind wanted to know everything about the protein so that he could decipher the mechanism by which protons were picked up on the cytoplasmic side of the membrane and pumped to the extracellular side of the membrane. This question was at the intellectual core of almost all of the systematic work carried out over more than 2 decades in his lab on bacteriorhodopsin, work that was performed by nearly 100 postdoctoral fellows and students and by dozens of collaborators.

At first, Gobind set out to determine the sequence of the protein by chemical means. This heroic feat was in close competition with Ovchinnikov’s group in Moscow and led to one of the first descriptions of how bacteriorhodopsin spanned the membrane seven times with seven hydrophobic stretches. These analyses laid the foundation for what is now a thriving field in the study of hepta-helical G-protein-coupled receptors in thousands of laboratories across academia and industry. With the knowledge of the amino acid sequence in hand, Gobind then initiated a number of experiments to ensure that the protein’s function in light-driven proton transport could be measured in the laboratory. This work required reconstitution of bacteriorhodopsin into lipid membranes, an area that was not familiar to him. Undaunted, he learned what was necessary for these experiments first-hand from a visit to Efraim Racker’s laboratory and launched a systematic set of experiments at MIT to dissect the function of the protein with his unique chemical approach.

In the 1980s, the thinking about how bacteriorhodopsin worked as a proton pump was heavily influenced by the “proton wire” concept, championed among others by Manfred Eigen. This theory was based on the idea that the passage of a proton across the membrane would be similar to proton conduction in ice where long-range movements of protons are achieved by a relay of protons along an extended hydrogen-bonded network. While physicists and physical chemists published papers exploring the merits of this idea, Gobind set out to test this idea with a strategy that bore his signature approach to molecular biology. He decided he would take out every possible residue in the protein that was capable of forming a hydrogen bond, one at a time, by site-specific mutagenesis and then test each of these mutants functionally for protein transport. His idea was that once the identity of all of the residues that influenced proton pumping activity was determined, they would reveal the secret of the path of the proton across the membrane.

This was a bold and grand undertaking. To execute this plan, every serine, threonine, tyrosine, aspartic acid, and glutamic acid had to be replaced, one at a time, by closely matched non-polar residues. No one had undertaken anything on this scale in protein structure–function studies at that time. PCR methods, which were first pioneered in Gobind’s laboratory, were still in their early days, but he did not like the prospect of even the small error rates introduced by Taq polymerase that could result in unexpected mutations. The only way to do this rigorously was to synthesize the entire gene and change out the residues one at a time by Gobind’s preferred approach of cassette mutagenesis. Methods had to be developed to efficiently express the mutants, purify them, and test them functionally. There was no one in the world who knew more about total gene synthesis than Gobind, and thus began the massive work to mutagenize bacteriorhodopsin.

Hundreds of single, double, and triple mutants were made with astonishing efficiency, and these were characterized not just at MIT but in numerous collaborating laboratories, all aimed at understanding the effects of each one of these mutations. Gobind’s bold and systematic approach shattered the notion that a project on this scale was too difficult. As the biochemical, biophysical, and structural studies of the mutants began to pour in in the late 1980s and early 1990s, the mystery of how bacteriorhodopsin actually worked gradually emerged. Interestingly enough, it turned out it was not really a proton wire in the way that Eigen had imagined. There were just a few residues, Asp 85, Asp 212, and Asp 96, that appeared to hold the keys to the mechanism of the proton pumping by providing alternating access to the Schiff base between retinal and Lys 216 in the middle of the membrane. In the end, it turned out to not really be a wire, but instead, a dynamic protein that uses selective movements of retinal initiated by light to trigger conformational changes that switched access of the central Schiff base to one or the other side of the membrane.

Closer observers of bacteriorhodopsin may take issue with my very selective summary of progress reported in thousands of papers in the bacteriorhodopsin field over nearly half a century! Needless to say, there was work in numerous other laboratories that led to seminal advances in the understanding of the mechanism by which proteins like bacteriorhodopsin worked. Contributions from leaders in the field such as Dieter Oesterhelt, Richard Henderson, Maarten Heyn, Ken Rothschild, Janos Lanyi, Vladimir Skulachev, Koji Nakanishi, and many others were essential in unraveling the fundamental mechanisms underlying the function of bacteriorhodopsin. And today, rapid advances in structural biology of membrane proteins have led to the determination of atomic resolution structures of hundreds of membrane proteins that can be studied functionally in much more precise detail than these early studies with bacteriorhodopsin. My point, however, is to merely highlight how the bread crumbs that were set out by Gobind provided a road map for future structure–function studies of membrane proteins and discoveries that were not connected to his initial mission. Who could have predicted that his work on bacteriorhodopsin would lead the way to the field of optogenetics that is now an incredibly powerful way to introduce light-activated genes into organisms, map the functional connectivity of the brain, and control the firing of neurons?

Gobind’s legacy in science is enormous. There is not a single laboratory in the world that has not been impacted by the advances Gobind pioneered for gene synthesis or in the systematic approach he established for structure–function studies of proteins. We take for granted that we can simply “place an order for a gene online” and have it show up in the lab. Five decades ago, this is the “impossible” feat that Gobind showed was possible with the complete synthesis of a gene for tRNA. The phrase “PCR test” is now familiar to billions across the world in the context of testing for COVID-19. A decade before the formal invention of PCR, it was Gobind’s group that showed how successive rounds of hybridization and DNA synthesis could be carried out with polymerase to make more of the desired DNA product without any additional labor in chemical synthesis of DNA. And his commanding approach to dissect the molecular mechanisms underlying the function of proteins such as bacteriorhodopsin and mammalian rhodopsin is still relevant today. Gobind’s record of his adventures in science is captured in a fascinating compendium that he put together toward the end of his career, tracing his path through some of the key ideas that shaped the science he carried out over many decades (Khorana 2000).

Gobind was born and brought up in a little village in Punjab. How did this scientist from such humble origins end up making such a profound impact on science for over 7 decades, with societal impact of his work continuing undiminished to this day? At Gobind’s 70th birthday celebration in Vancouver in 1993, one of his former associates compared his stature in science to that of the Alps: tall and unyielding, with tremendous span and an everlasting and towering presence. Add to that single-minded focus, relentless tenacity, great discipline, unforgiving pursuit of the highest standards in scientific integrity and accuracy, genuine humility, and an unending thirst for knowledge, and perhaps that begins to give us just a small glimpse of what made Gobind such an incredible force of nature in the world of biological sciences.
